# Pillar-beam structures prevent layered cathode materials from destructive phase transitions

**DOI:** 10.1038/s41467-020-20169-1

**Published:** 2021-01-04

**Authors:** Yuesheng Wang, Zimin Feng, Peixin Cui, Wen Zhu, Yue Gong, Marc-André Girard, Gilles Lajoie, Julie Trottier, Qinghua Zhang, Lin Gu, Yan Wang, Wenhua Zuo, Yong Yang, John B. Goodenough, Karim Zaghib

**Affiliations:** 1 Center of Excellence in Transportation Electrification and Energy Storage, Hydro Québec, 1800 Boulevard Lionel-Boulet, Varennes, Québec, J3X 1S1 Canada; 2 Key Laboratory of Soil Environment and Pollution Remediation, Institute of Soil Science, Chinese Academy of Sciences, Nanjing, Jiangsu 210008 China; 3 Laboratory of Advanced Materials and Electron Microscopy, Beijing National Laboratory for Condensed Matter Physics, Institute of Physics, Chinese Academy of Sciences, Beijing, 100190 China; 4 Advanced Materials Lab, Samsung Research America, Cambridge, MA 02138 USA; 5 State Key Laboratory for Physical Chemistry of Solid Surfaces, and Department of Chemistry, College of Chemistry and Chemical Engineering, Xiamen University, Xiamen, 361005 China; 6 The University of Texas at Austin, Austin, Texas 78712 USA

**Keywords:** Batteries, Batteries, Batteries

## Abstract

Energy storage with high energy density and low cost has been the subject of a decades-long pursuit. Sodium-ion batteries are well expected because they utilize abundant resources. However, the lack of competent cathodes with both large capacities and long cycle lives prevents the commercialization of sodium-ion batteries. Conventional cathodes with hexagonal-P2-type structures suffer from structural degradations when the sodium content falls below 33%, or when the integral anions participate in gas evolution reactions. Here, we show a “pillar-beam” structure for sodium-ion battery cathodes where a few inert potassium ions uphold the layer-structured framework, while the working sodium ions could diffuse freely. The thus-created unorthodox orthogonal-P2 K_0.4_[Ni_0.2_Mn_0.8_]O_2_ cathode delivers a capacity of 194 mAh/g at 0.1 C, a rate capacity of 84% at 1 C, and an 86% capacity retention after 500 cycles at 1 C. The addition of the potassium ions boosts simultaneously the energy density and the cycle life.

## Abstract (web summary)

The specific capacity of P2-type sodium-ion battery cathode is limited because full extraction of Na ions leads to structural degradation. Here authors report pillar-beam structured material to overcome this issue by using K pillar ions to uphold the transition metal layers upon extraction of Na ions.

## Introduction

Lithium-ion batteries with high energy density and long cycle life have become the dominant energy storage technology in electronic devices and electric vehicles. However, owing to the limited lithium and cobalt resources and consequently their rising costs, relying solely on lithium-ion batteries to meet the rapidly increasing demand for energy storage presents a great risk^1^. Sodium-ion batteries (SIBs) have been considered as a promising alternative to lithium-ion batteries owing to the abundance of sodium on the Earth and to the use of lower-cost transition metal elements in the electrodes. They could play a critical role in large-scale stationary renewable energy systems where lifetimes and availabilities are most desirable^2–7^.

**Fig. 1:**
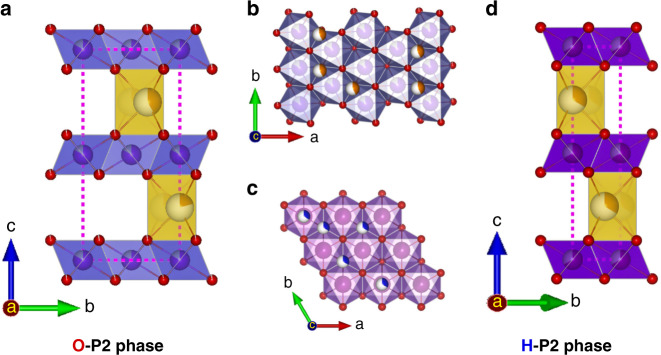
Crystal structure of O-P2 phase and H-P2 phase. Schematic illustration of the orthorhombic P2 phase projected in (**a**) *b–c* plane, (**b**) *a–b* plane or along *c*-direction. And H-P2 phase in (**c**) *a–b* plane, and (**d**) *b–c* plane. Brown balls represent potassium, red balls oxygen, and blue balls transition metal atoms. Partially colored balls represent partial occupancy where the portion of white area represent the likelihood of the site's not having an atom.

A generally recognized challenge to the development of SIBs is to simultaneously improve the capacity and the cycle life of the sodium-ion cathode. Among the many potential cathode materials^8–13^, layered metal oxides have many advantages because their layered structures provide large capacity, high rate capabilities, and have a relatively lower manufacturing cost^14–21^. These layered transition metal oxides can be categorized into P2, P3, O2, and O3 types according to the oxygen alignment around the Na^+^ ions and the stacking of the transition metal oxide layers^22^. O3-type materials include the commonly used LiCoO_2_ and NMC (nickel–manganese–cobalt) oxides in current lithium-ion batteries^23,24^. Despite the success of these O3 materials in lithium-ion battery cathodes, when used in SIBs, NaCoO_2_ and their derivatives undergo complicated phase transitions during discharge O3 → O'3 → P3 → P'3 → P″3, and the materials fail rapidly if the content of sodium falls below 50–60%, resulting in a limited capacity of ~110 mAh/g^25–27^. P2-type structures have two possible symmetry groups, as shown in Fig. 1. One is the hexagonal P2 (H-P2) with symmetry group P63/mmc, which is the most conventional symmetry group of P2 materials. The other possibility is orthogonal P2 (O-P2) whose symmetry group is Cmcm; there is currently no direct use of O-P2 structures in the cathode materials for SIBs, to the best of our knowledge. H-P2-type structures have fewer phase transitions than those of O3, but the P2 → O2 transition at ~33% sodium content still leads to material failure and the capacity is severely capped by this high lower-bound of sodium. One exception to this lower-bound is where lithium is inserted in the transition metal layers of H-P2-type Na_0.72_[Li_0.24_Mn_0.76_]O_2_, whose first-cycle capacity can reach 210 mAh/g (full extraction of sodium ions). However, during cycling, the anions are involved in the charge transfer and hence oxygen evolution reactions occur, leading to poor stability^28,29^.

Solutions have been previously proposed to improve either the stability or the capacity of the P2-type SIB cathode materials, but never both. High stability requires that the anions do not participate in the charge transfer; high capacity requires that the range of sodium content during the battery operation is as wide as possible. Therefore, on the one hand, one could try to increase the sodium content of the materials during synthesis so that the battery could operate in a wider range of sodium content (upper-bound elevated) without costly preprocessing; on the other hand, one could also try to suppress the phase transition at 33% of Na so that the lower-bound of operational sodium content is lowered, allowing for a wider range of sodium and consequently leading to a higher capacity. There is not much room for the improvement with the first approach because, when the sodium content in H-P2-type oxides is more than 80% or less than 60%, the material is not chemically stable in the air^30^. Preventing its reaction with the air would require a lot of extra work and greatly increase the manufacturing cost. Regarding the latter approach, surface coating and doping in the transition metal layer have been tried^31–33^. Neither of these methods could suppress a phase transition or prevent material failure caused by such transitions.

We envisage the fact that such phase transitions are inevitable for the moment and seek alternative approaches that might be more effective. We, therefore, did not aim to suppress the phase transition, but to postpone the phase transition to well below the “33%” threshold and to  maintain a stable structural framework during the transition. We tackled the problem not by doping the transition metal layer, but by doping the alkali ion layer. In this study, we present this “pillar-beam” structure with the formula K_0.08_Na_[0-0.74]_[Ni_0.2_Mn_0.8_]O_2_ (K_0.4_[Ni_0.2_Mn_0.8_]O_2_ before cycling). In this material, a small percentage of potassium ions are “inert” during the cycling and stay in the alkali ion layer permanently, thus stabilizing the structure. Similar strategies have been applied to lithium-ion cathode materials in previous reports^34–36^, where dopants in the alkali ion layers are shown to help prevent the formation of spinel phase at the surfaces. Nevertheless, no single phase material was obtained in these lithium cathodes, but here we managed to synthesize an O-P2 phase powder for the Na cathode. In addition, the phase transition prevention is extended from the surface to the bulk in the current work. More importantly, the detailed underlying mechanism of the dopant ions is fully presented. We demonstrate that the doping potassium ions, which are larger than sodium, support the transition metal layers even while the sodium ions are completely extracted (hence the term “pillar-beam”). The P2 → O2 phase transition only occurs at nearly the end of sodium extraction, so that it no longer destroys the cathode materials and has little effect on the cyclability. By replacing around 8% of the sodium ions with potassium ions, we bypassed the 33% sodium content limit, thus benefiting from an extra 25% of sodium ions for the intercalation reaction. If a non-doped material works in the sodium content range of 33–80%, then we can increase the capacity by ~50% with the potassium “pillars”.

In this work, we report an orthogonal P2-type structured cathode material K_0.4_Ni_0.2_Mn_0.8_O_2_. It delivers a high capacity of 194 mAh/g at 0.1 C, a high rate capacity of 84% at 1 C, and an 86% capacity retention after 500 cycles at 1 C. In situ X-ray diffraction (XRD) spectra, inductively coupled plasma (ICP) results, and scanning transmission electron microscopy (STEM) images demonstrate that the remaining potassium ions preserved the pillar-beam framework even when all the sodium ions are extracted. X-ray absorption fine structure (XAFS) spectra and differential electrochemical mass spectrometry (DEMS) also show that it is the transition metal ions, rather than the oxygen, that are involved in the charge transfer during the cycling period. The first-principles density functional theory (DFT) calculations provide insights into the diffusion properties of sodium and potassium ions, further supporting the hypothesized function of potassium ions in the material. The study of the detailed working mechanism of the potassium pillar ions in the alkali layer reveals their effect in boosting both the energy density and the cycle life.

## Results

### Structure and sodium storage of K_*x*_[Ni_*x*/2_Mn_1*−x*/2_]O_2_

**Fig. 2:**
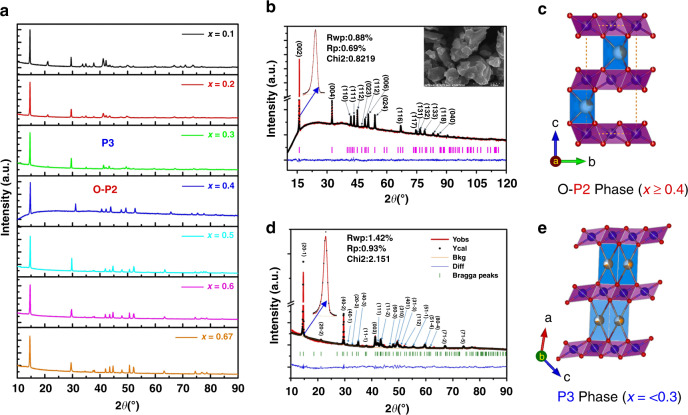
Structure of K_*x*_Ni_*x/2*_Mn_1*−x/2*_O_2_. **a** XRD patterns of K_*x*_Ni_*x*/2_Mn_1−*x*/2_O_2_ compounds prepared by solid state reaction, arranged in order of increasing potassium content from top to bottom (*λ* = 1.789 Å). **b** The Rietveld refinement for the K_0.4_Ni_0.2_Mn_0.8_O_2_ XRD pattern. **c** Schematic illustration of the orthorhombic P2-K_0.4_Ni_0.2_Mn_0.8_O_2_ projected in *b–c* plane. **d** The Rietveld refinement for the K_0.3_Ni_0.15_Mn_0.85_O_2_ XRD pattern. **e** Schematic illustration of the Monoclinic K_0.3_Ni_0.15_Mn_0.85_O_2_ projected in *a*–*c* plane.

A series of compounds, specifically K_*x*_[Ni_*x*/2_Mn_1*−x*/2_]O_2_ with *x* = 0.1, 0.2, 0.3, 0.4, 0.5, 0.6, and 0.67, were synthesized to study the effect of potassium content on the crystal structure. Although they were all layered, the Rietveld refinements of their XRD patterns showed that these compounds could be categorised into two symmetry groups, as shown in Fig. 2a. When *x* ≥ 0.4, they are O-P2 type. This O-P2 structure belongs to the orthorhombic crystal system with symmetry group Cmcm, where potassium ions occupy the 4c sites (see Supplementary Table 1 for the detailed atom locations). The potassium ions are contained in the triangular prisms sharing their base edges with those of the transition metal octahedral, and if viewed along the *c*-axis, the potassium ions are centered in triangles with transition metal atoms at the vertices. This is different from the conventional H-P2-type structure with symmetry group P63/mmc where, in addition to edge-sharing 2d sites, part of the alkali-metal ions occupied face-sharing 2b sites with transition metal atoms directly on top of them, as shown in Fig. 1. The O-P2 structure with *x* = 0.4 is plotted in Fig. 2c; its lattice parameters are *a* = 2.886(5) Å, *b* = 5.001(7) Å, *c* = 12.7908 Å, and *V* = 184.666 Å^3^. When *x* ≤ 0.3, however, these main phases become P3 which are monoclinic with symmetry group C2/m, where potassium ions partially occupy the 4i sites (Supplementary Table 1a, b). The exemplifying *x* = 0.3 case is plotted in Fig. 2d, e; its lattice parameters are *a* = 5.118(7) Å, *b* = 2.860(3) Å, *c* = 7.181(0) Å, and *V* = 105.012 Å^3^.

**Fig. 3:**
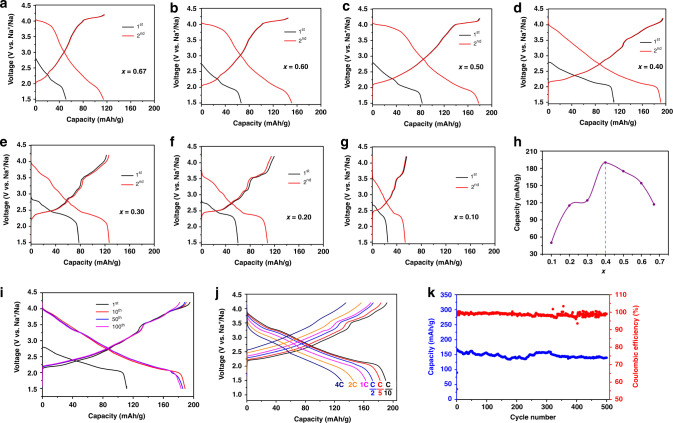
Sodium storage performance of K_*x*_Ni_*x/2*_Mn_1−*x/2*_O_2_ electrodes. The 1^st^ and 2^nd^ discharge/charge curves at C/10 between 1.5 and 4.2 V versus Na^+^/Na for the material **a** K_0.67_Ni_0.33_Mn_0.67_O_2_, **b** K_0.6_Ni_0.3_Mn_0.7_O_2_, **c** K_0.5_Ni_0.25_Mn_0.75_O_2_, **d** K_0.4_Ni_0.2_Mn_0.8_O_2_, **e** K_0.3_Ni_0.15_Mn_0.85_O_2_, **f** K_0.2_Ni_0.1_Mn_0.9_O_2_, **g** K_0.1_Ni_0.05_Mn_0.95_O_2_. **h** Comparison of the 2^nd^ discharge capacity of K_*x*_Ni_*x/2*_Mn_1−*x/2*_O_2_ versus *x*. Sodium storage performance of O-P2 K_0.4_Ni_0.2_Mn_0.8_O_2_ electrodes: **i** The 1^st^, 10^th^, 50^th^, and 100^th^ discharge/charge curves at C/10 between 1.5 and 4.2 V versus Na^+^/Na. **j** Rate capability. The capacity versus cycle number at various current rates. **k** Long-term cycling performance. The capacity and Coulombic efficiency versus cycle number at 1 C. Note that the K content stabilized at 0.08 after first cycle, therefore, the working electrode was Na_*x*_K_0.08_Ni_0.2_Mn_0.8_O_2_ thereafter.

The electrochemical results of K_x_[Ni_x/2_Mn_1-x/2_]O_2_ | Na cells (*x* = 0.1, 0.2, 0.3, 0.4, 0.5, 0.6, 0.67) are shown in Fig. 3a–h, where all the cells were first discharged to 1.5 V, then charged to 4.2 V, and finally discharged again. To summarize the results: (1) the *x* = 0.4 case had a maximum capacity for the second discharge; and (2) all the materials’ second discharge showed more capacity than their respective first discharges. The latter feature was readily understood as the result of the partial replacement of potassium ions by sodium ions. After the first discharge, sodium ions entered the structure via both ion-exchange and electrochemical intercalation; they could be extracted during the first charge, leaving more vacancies for the next discharge. But at least for the optimum case of *x* = 0.4, even potassium ions could get further extracted during the first charge, leaving even more available sites for sodium, and although all the cells reached equilibrium at an open-circuit voltage (OCV), the potassium equilibrium under the electrochemical conditions was reached only after the first cycle. We also discuss this below.

Since the *x* = 0.4 material K_0.4_Ni_0.2_Mn_0.8_O_2_ has the best electrochemical performance, further investigations were focused on this material only. Detailed cycling performances are shown in Fig. 3i–k. The first discharge exhibited a capacity of 115 mAh/g between 2.85 and 1.5 V versus Na/Na^+^. This capacity corresponds to a 0.441 sodium-ion intercalation per formula unit; at this moment, the total amount of alkali-metal ions becomes 0.841 per formula unit, which includes the initial 0.4 potassium (some of them being replaced by sodium due to the ion-exchange effect) and the 0.441 intercalated sodium. At the immediate next charging state, we see that the reversible capacity was 194 mAh/g between 1.5 and 4.2 V, corresponding to 0.748 alkali ion extraction. Hence, only 0.093 alkali ions were left in the cathode material at the end of the charge (before the second discharge). The ICP results suggest that at this stage there are 0.0991 K ions left in the material (see Table 1); therefore, we are certain that the 0.093 alkali ions that were left here are all K ions and that the 0.748 extracted alkali ions contained both K and Na. Note that ICP tests are destructive so different cells are used, and the derived values of 0.0991 and 0.093 K ions can be well considered to be consistent. The almost vacant alkali-metal ion layer led to high capacity in the following cycles. In the second cycle, this material appeared quite stable; the charge/discharge curves were barely changed. At a current density of C/10, excellent capacity retention of 95.6% was achieved at the 100^th^ cycle. It is worth noting that, unlike conventional H-P2-type cathode materials, there was no plateau in the charge curve for sodium content below 33%. This observation suggests that no serious side reactions, such as gas evolution reactions, took place during the charging stage. After ion-exchange, the escaped K ions enter and stay in the electrolyte in the form of KPF_6_ even with metallic Na anodes (see supplement information for the details, shown in Supplementary Figs. 1–4). So the pre-treatment of ion-exchange does not trigger any safety issues in the cells. The rate capability of this O-P2-type K_0.4_[Ni_0.2_Mn_0.8_]O_2_ electrode was also tested. It can be seen in Fig. 3j that the reversible capacities were 194, 183, 174, 163, 147, and 135 mAh/g at current rates of C/10, C/5, C/2, 1 C, 2 C, and 4 C, respectively. The capacity retention at 1 C was 84% of the initial capacity. The capacity retention was 86% at 1 C after 500 cycles (shown in Fig. 3k). The Coulombic efficiency (CE) was nearly 100% for almost all cycles. Some fluctuations in the CE curve in Fig. 3k (upper curve) are observed. Many factors can contribute to cell fluctuations, including temperature, humidity, stress, electrolyte reactions, and SEI formations^37,38^. A few solutions have been proposed^39^, but fluctuations remain inevitable. Thus, with the help of the O-P2 pillar-beam structure of K_0.4_[Ni_0.2_Mn_0.8_]O_2_, a large capacity and high stability in this SIB cathode material were achieved simultaneously. In this *x* = 0.4 case, after first cycle, the content of K is stabilized at 0.08 and it is indeed Na_*x*_K_0.08_Ni_0.2_Mn_0.8_O_2_ at work in what follows.

**Table 1:** ICP results of cathode material at indicated states.

State	K	Ni	Mn (normalized)
Pristine	0.4032(3)	0.2063(2)	0.8
OCV	0.2054(7)	0.2052(7)	0.8
1^st^ disch. −2.35 V	0.1512(6)	0.2034(2)	0.8
1^st^ disch. −1.5 V	0.1349(8)	0.2005(4)	0.8
1^st^ ch. −2.7 V	0.1110(5)	0.2035(1)	0.8
1^st^ ch. −3.3 V	0.1058(7)	0.2039(8)	0.8
1^st^ ch. −4.2 V	0.0991(0)	0.1992(3)	0.8
2^nd^ disch. −3.4 V	0.0888(3)	0.2017(5)	0.8
2^nd^ disch. −1.5 V	0.0835(4)	0.2051(9)	0.8
2^nd^ ch. −4.2 V	0.0816(3)	0.2025(8)	0.8
3^rd^ disch. −1.5 V	0.0828(4)	0.2070(5)	0.8
3^rd^ch. −4.2 V	0.0846(9)	0.2052(7)	0.8
5^th^ disch. −1.5 V	0.0845(8)	0.1991(8)	0.8
5^th^ ch. −4.2 V	0.0824(6)	0.2050(4)	0.8
10^th^ disch. −1.5 V	0.0828(4)	0.2004(1)	0.8
10^th^ ch. −4.2 V	0.0839(6)	0.2044(5)	0.8

Note: “ch.” is charge. “disch.” is discharge.

### Charge transfer and structural changes

Several factors contributed to the excellent capacity and stability of K_0.4_[Ni_0.2_Mn_0.8_]O_2_ (which becomes Na_*x*_K_0.08_Ni_0.2_Mn_0.8_O_2_ after the first cycle). It was known that phase transitions and gas evolution were major reasons for the limited capacity and stability of conventional SIB cathode materials. In this section, we discuss how our material avoided these pitfalls. Detailed examinations of the atomic charge transfers and the microscopic structures were conducted to understand the electrochemical behavior of K_0.4_[Ni_0.2_Mn_0.8_]O_2_ (Na_x_K_0.08_Ni_0.2_Mn_0.8_O_2_). It was found that the anions in this K_0.4_[Ni_0.2_Mn_0.8_]O_2_ (Na_x_K_0.08_Ni_0.2_Mn_0.8_O_2_) did not participate in any chemical or electrochemical reactions and the conventionally destructive P2 → O2 phase transition occurred only towards the end of the sodium extraction.

**Fig. 4:**
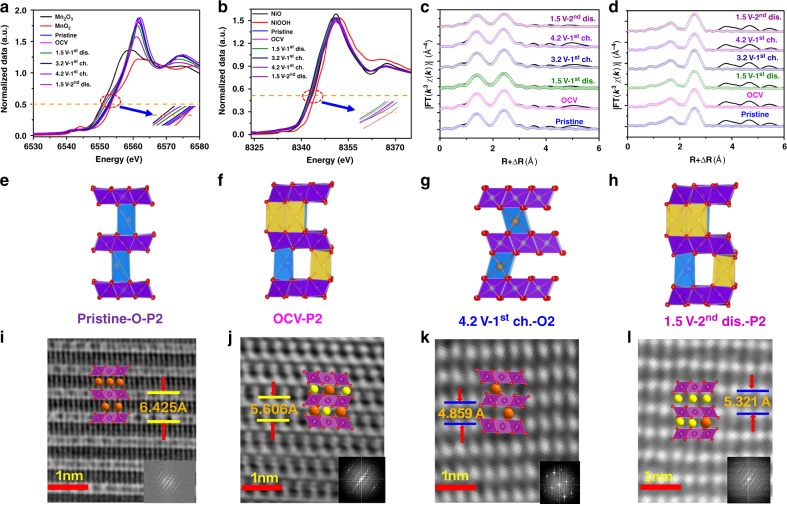
Charge transfer and STEM images of O-P2 K_0.4_Ni_0.2_Mn_0.8_O_2_ during cell operation. **a** Mn and **b** Ni K-edge ex situ XANES spectra collected at indicated states. Spectra of reference materials were presented for comparison. **c** Shell-by-shell fitting results for the Fourier-transformed EXAFS of Mn and **d** Ni in the same states. The most energetically favorable structures suggested by DFT calculations at the state of **e** Pristine, **f** OCV, **g** 4.2 V-1^st^ charge, and **h** 1.5 V-2^nd^ discharge. These results match well with the following STEM images: **i** ABF-STEM image of pristine K_0.4_Ni_0.2_Mn_0.8_O_2_, **j** OCV, and HAADF-STEM image of K_0.4_Ni_0.2_Mn_0.8_O2 (H-P2) at the state of **k** 4.2 V-1^st^ charge and **l** 1.5 V-2^nd^ discharge.

The destructive gas (oxygen) evolution reaction, a threat to the stability of SIB cathode materials, typically comes from the oxidation of O^2−^ anions in the structure. Detection of O^2−^ was conducted by monitoring the valence changes of all elements by using X-ray absorption near edge structure (XANES) spectroscopy and operando differential electrochemical mass spectroscopy (DEMS) during battery cycling. XANES spectra revealed that the Mn edge was between those of Mn_2_O_3_ (Mn^3+^) and MnO_2_ (Mn^4+^), indicating that its average valence was between +3 and +4, and that the Ni edge was between NiO and NiOOH, indicating that its average valence was between +2 and +3 (Fig. 4a, b). Supplementary Table 2 shows the average Mn valences at each charge state, according to the linear regression between the absorption edge and the voltage of standards (Supplementary Fig. 5). During the first discharge, the voltage dropped from 2.7 to 1.5 V and the valence of Mn decreased from +3.65 to +3.10, which indicates that 0.44 Mn atoms changed from +4 to +3 per formula unit (as there are 0.8 Mn atoms in a formula unit); and compared to the number of sodium ions (0.441) that intercalated during this period, we see that it is the sodium intercalation in this voltage range that led to the corresponding Mn reduction. Upon charging the cell to 3.3 V, the Mn valence increased to +3.70, demonstrating good redox reversibility. Further charging the cell to 4.2 V led to an increase in Mn valence to +3.95; therefore, from 1.5 to 4.2 V, the charge transfer reflected by the Mn valence was determined to be 0.68 electrons per formula unit. Meanwhile, no significant changes in the Ni absorption edge were observed during the first discharge (from 2.7 to 1.5 V). However, during the charge from 1.5 to 4.2 V, the absorption edge has shifted 0.6 eV upwards. This upward shift of the edge is known to represent a valence change of +0.3 for Ni, according to the linear fitting with the absorption edge of NiO (8345.8 eV) and NiOOH (8347.8 eV). Given that there were 0.2 Ni atoms per formula unit, the overall charge was 0.06 electrons per formula unit. Oxygen valence did not change during the electrochemical reaction, as shown by the oxygen K-edge near-edge X-ray absorption fine structures (NEXAFS) results (see Supplementary Fig. 6). Therefore, the total charge transfer per formula during the charge from 1.5 to 4.2 V was 0.68 + 0.06 = 0.74 electrons, while the determined total number of the sodium ion extraction was 0.748; the difference between the two is 1.08%. The DEMS measurement for this process is shown in Supplementary Fig. 7. No signal of O_2_ gas has been detected during the whole charge–discharge process. This is consistent with the results from NEXAFS spectroscopy. Meanwhile, a small amount of CO_2_ gas was released. We believe it was generated by the decomposition of traces of Na_2_CO_3_ in the electrode and the side reactions involving the electrolyte at high voltage^40–42^. To conclude, based on these results that the charge transfer during the cell operation led to valence changes of the transition metal ions only, and oxygen ions did not participate in the electrochemical reaction, hence no gas evolution reactions occurred. This explains the observed high stability of K_0.4_[Ni_0.2_Mn_0.8_]O_2_/Na_x_K_0.08_Ni_0.2_Mn_0.8_O_2_. In addition, where Mn and Ni were both present within the lattice, it was determined that Mn underwent a valence change before Ni. Identical results to the first cycle were obtained in the second cycle, demonstrating the excellent reversibility and stability of this material.

Having ruled out the possibility of gas evolution reactions, the structural changes accompanying the cycling process were investigated using multiple techniques. ICP was used to determine the quantity of the “pillar” potassium. XAFS spectra were used to investigate the local structure and an overall lattice evolution was probed via XRD. STEM images were then acquired to examine the microstructure of K_0.4_[Ni_0.2_Mn_0.8_]O_2_. Finally, first-principles DFT calculations were performed to further understand the energetics of the phases involved. All these techniques convinced us that our material was highly stable during the cycling; the P2 → O2 phase transition was postponed till nearly the end of the sodium extraction, and it did not interfere with the intercalation reaction or did not result in any structural damage.

The ICP results are shown in Table 1. The potassium content initially decreased due to ion-exchange and further decreased during the cycling period, as discussed earlier. However, after the first cycle, the K content remained constant, suggesting that equilibrium was reached. These “pillar” potassium ions occupied ~8% alkali metal sites, thus leaving an extra 25% of alkali metal sites available for sodium intercalation/de-intercalation. Consequently, the lower-bound of Na content is reduced from 33% in the conventional H-P2-type materials to ~8% in this O-P2-type material, which accomplished the high capacity.

Fourier-transformed extended X-ray absorption fine structure (EXAFS) analyses were employed to examine the local structural changes during the electrochemical cycle. The shell-by-shell fitting results (Supplementary Table 3a, b and Fig. 4c, d) showed high stability of the material during the charge–discharge cycle from 1.5 to 3.4 V. The (averaged) Mn–O bonds have two different lengths of 1.86 and 1.99 Å due to the Jahn–Teller effect. Both bond-lengths remained unchanged. And so did the Mn–Mn/Ni distance of 2.88 Å. The Mn–O bond splitting in Jahn–Teller distorted MnO_6_ polyhedral is also confirmed in the first-principles DFT calculations (Supplementary Fig. 8). The coordination numbers of Mn to O and Mn/Ni elements had approximate values of 5.9 and 7.7, respectively. Substantial changes occurred during the charging process to 4.2 V, including the Mn–O bond length and Mn–Mn/Ni distance, which decreased to 1.83, 1.95, and 2.85 Å, respectively. This refers that there was a phase transition from P2 to O2 during this stage. The local structure around the Ni atoms did not exhibit significant changes.

The four states (pristine, OCV, 1^st^ 4.2 V-charge, and 2^nd^ 1.5 V-discharge) of the active materials were further analyzed at atomic resolution with annular bright field (ABF) and high-angle annular dark field (HAADF) STEM (shown in Fig. 4). Fig. 4e–h displays the most energetically favorable structures in each state, based on the DFT calculations. ABF-STEM images of pristine and OCV K_0.4_Ni_0.2_Mn_0.8_O_2_ are shown in Fig. 4i, j, respectively. They correspond to the orthogonal and hexagonal layered-structure phases with the inter-layer distances labeled. Fig. 4k, l exhibit the HAADF-STEM observation of the 4.2 V-1^st^ charge and 1.5 V-2^nd^ discharge state. The measured structures and inter-layer distance also aligned closely with our DFT calculations (see Supplementary Table 4).

**Fig. 5:**
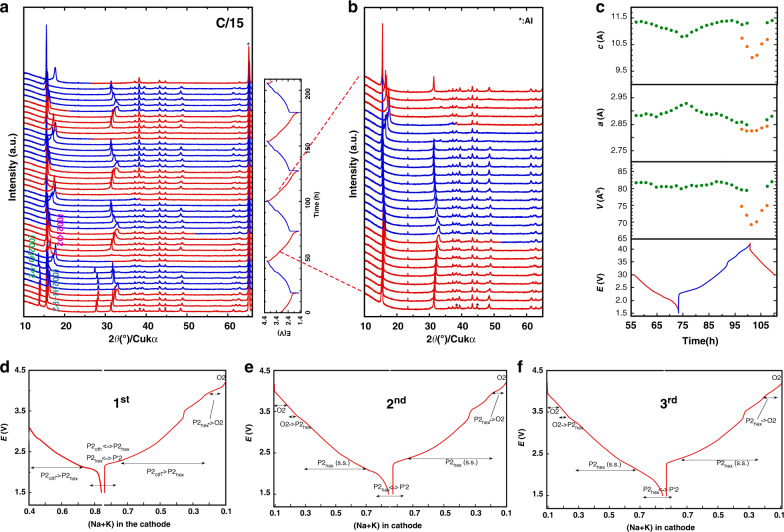
In situ XRD patterns of K_0.4_Ni_0.2_Mn_0.8_O_2_ electrode during electrochemical operation. **a** Selected XRD spectra collected during four cycles at C/15 between 1.5 and 4.2 V.; **b** XRD spectra focused on the 2^nd^ cycle; **c** Variation of lattice parameters during the 2^nd^ cycle; **d**–**f** Phase diagrams of 1^st^, 2^nd^, and 3^rd^ cycles, respectively.

In fact, before the electrochemical cycling, part of the O-P2 structure in the as-made cell has already converted to H-P2 via ion-exchange. The *c*-axis in the H-P2 phase (OCV state) was 11.417(7) Å as obtained from XRD pattern, which was close to the value observed from STEM (11.212 Å) (Fig. 4j). The crystal structure evolution during the cycling was studied via in situ XRD (Fig. 5). In the first cycle, O-P2 and H-P2 phases co-existed initially. When the discharge proceeded to ~2.1 V (K^+^ + Na^+^ = 0.76), the (002)_H-P2_ peak started to broaden and stayed till the end of the discharge period, along with an up-shift. The formation of an intermediate P’2 phase (orthorhombic, S.G.: Cm)^43–45^ was implied by the split of the (002) and (004) peaks and the emergence of another peak at ∼37° at the beginning of the charge, which was repeatedly observed in the following three cycles. This P′2 phase was formed by the collective gliding of transition metal layers with Na^+^ intercalation or with the distortions caused by the increasing amount of Mn^3+^ ^44–46^. While charged to ~4.0 V (∼0.162 Na^+^), the (002)_O-P2_ peak disappeared and a noticeable peak formed at ~16.6°, which was attributed to the (002) of O2 phase with a *c*-axis of 10.672 Å. This O2 phase was formed via the gliding of the transition metal layers and, as a result, the alkali metal sites changed from prismatic to octahedral. Further charging the cell to 4.2 V, the (002)_H-P2_ peak disappeared while the (002)_O2_ peak rapidly shifted to 17.8°, corresponding to the *c*-axis of 9.968 Å. This parameter was in agreement with the 9.718 Å that was captured in the STEM image (a 2.6% difference). The detailed information regarding the phase transformations during the first cycle is summarized in Fig. 5d. After the first cycle, the O-P2 phase disappeared completely with only the stable H-P2 phase left. This result could be due to the removal of potassium ions from 0.2 to 0.08 during the first charge, after which the potassium content kept constant. From the second cycle on, the spectrum development demonstrated excellent reversibility, with the H-P2 to O2 phase transformation at the end of charge. Some inflection points were observed on the charge/discharge curve. For example, at ~3.3 V (Na^+^ + K^+^ ~ 0.33), there was a small bump; further charging to ~3.5 V (Na^+^ + K^+^ ~ 0.5), there was a small drop. These subtle features on the electrochemical curves were due to Na^+^/vacancy ordering in the structure^47,48^, which had little effect on the main pillar-beam structure or the electrochemical performance.

The evolution of the lattice parameters of H-P2 and O2 phases in the 1^st^ and 2^nd^ cycles is shown in Supplementary Fig. 9. The variations during the two cycles are similar despite the existence of O-P2 phase in the 1^st^ cycle. Fig. 5c plots the lattice parameter changes in the 2^nd^ cycle. During the discharge period from 3 to 1.5 V, *c*_H-P2_ decreased continuously and reached a minimum at 1.5 V, whereas *a*_H-P2_ displayed the opposite trend. During the charging period, *c*_H-P2_ increased until O2 phase was present. The lattice parameter changes were Δ*c*_H-P2_ = 5.29%, Δ*a*_H-P2_ = 2.74%, Δ*V*_H-P2_ = 2.99% for the H-P2 and Δ*c*_O2_ = 6.81%, Δ*a*_O2_ = 0.6%, Δ*V*_O2_ = 7.55% for the O2 phase. This phase transition introduced a total volumetric change of 12.8% which is smaller than those of the conventional SIB cathode materials (~23.1%)^49^. This phenomenon revealed the important role of the potassium pillars in reducing the strain to a tolerable level and thus stabilizing the structure. The importance of potassium pillar ions can be further evidenced by the excessive voltage in charge. In a destructive test, we charged the cell to 4.5 V at the rate of C/15, the cell showed fast degradation (see Supplementary Fig. 10a). Supplementary Fig. 10b shows the voltage curve started to be noisy after a short smooth slope above 4.2 V, which is likely related to the contact issue. This short slope started at the 4.2 V and ended at 4.35 V. The capacity provided by this slope matched exactly the remaining 8% K ions. The lattice parameter *c* of the O2 phase in the 1^st^ cycle was calculated from the XRD pattern and plotted in Supplementary Fig. 10c, d. The *c*-axis of the O2 phase was 4.9794 Å at 4.2 V and reduced to 4.9730 Å by further charging the cell to 4.35 V. The decrease in *c* was caused by the removal of the 8% potassium ions from the structure. Although we can see from the XRD patterns that even under such conditions the layer structure could still be maintained, the material becomes highly irreversible regarding the intercalation reaction with Na ions and this proves the vital role of potassium ‘pillar’ ions in enhancing the performance of this material. In addition to propping the structure and reducing the strain during cycling, K ions also help to reduce the Na intercalation potential by stretching the transition metal layers so that they could not provide the optimum inter-layer distance for the intercalated Na (see Supplementary Fig. 11 and Discussion in Supplementary Information).

### Insights from first-principles calculations

**Fig. 6:**
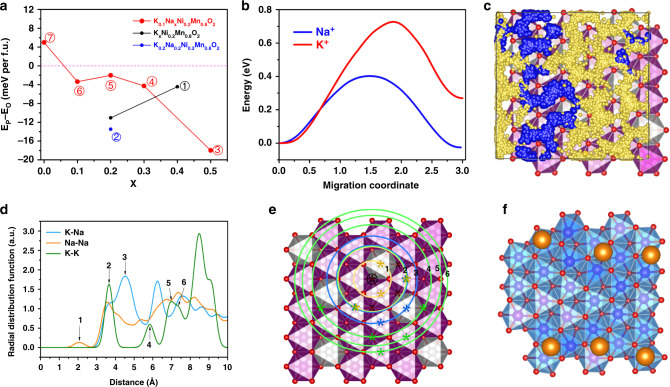
DFT calculations. **a** Energy difference between P2 and O2 phase of each composition found during the cell operation. **b** Na^+^ and K^+^ vacancy hopping energy barriers. **c** Trajectories of Na^+^/K^+^ ion diffusion in the alkali metal layer in P2- K_0.1_Na_0.3_Ni_0.2_Mn_0.8_O_2_ from AIMD simulations. **d** Radial distribution function (RDF) for the alkali ions.** e** Comparison between the RDF peak positions and the *a*–*b* plane of the crystal lattice. **f** Distribution of K ions after structural relaxation. Inter-K distances are all larger than the inter-site distance even if they are put closer at the beginning of the structural relaxation.

We also verified the condition of the P2 → O2 phase transition using first-principles DFT calculations. The energy difference between the P2 and O2 phases at different alkali metal contents are shown in Fig. 6a. The P2 phase is favored over O2 phase in the pristine K_0.4_Ni_0.2_Mn_0.8_O_2_ composition (marked “1” in black). After the ion-exchange, the resulting material K_0.2_Na_0.2_Ni_0.2_Mn_0.8_O_2_ (marked “2” in blue) was still energetically favored as the P2 phase. During the cycling period, the content of potassium changed to 0.1 (or 10%) and the content of sodium ranged from 0 to 0.7. All considered compositions of K_0.1_Na_*x*_Ni_0.2_Mn_0.8_O_2_ (marked “3”, “4”, “5”, and “6” in red) favored the P2 phase except when the sodium content drops to 0 (marked “7” in red), where the O2 phase became much more energetically favored. Therefore, the P2 → O2 phase transition only occurred when the sodium content dropped below 0.1. We should note that, even where this phase transition did occur, there were still 0.1 potassium ions in the alkali metal layer propping the structure. Later cycles did not result in changes in the potassium content. We believe there were two reasons for this relatively constant potassium content. First, the equilibrium redox potential of K/K^+^ is 0.2 V lower than that of Na/Na^+^; therefore the potassium ions could remain in the electrolyte while the sodium was deposited on the anode, leaving the equilibrium between K^+^ in the electrolyte and K^+^ in the cathode material largely unaffected. Second, the calculated energy barrier of K^+^ vacancy migration in the alkali layer was higher than that of Na^+^ in P2-K_0.2_Na_0.8_Ni_0.2_Mn_0.8_O_2_, as shown in Fig. 6b.

The larger K migration barrier is mainly due to the localization of K-ions in the structure, which creates a higher energy cost of hopping to an adjacent site. To characterize the overall hopping behavior, we adopt the notion of kinetically resolved activation (KRA) energy barrier obtained by subtracting from the usual activation energy the difference between initial and final state total energies. In this way, an average difference of 0.18 eV in KRA energy barriers between K^+^ and Na^+^ at room temperature translates to a difference in hopping probability of orders of magnitude. Furthermore, ab initio molecular dynamics (AIMD) simulation was performed to investigate the diffusion of alkali-metal ions in the P2 phase. Fig. 6c shows the trajectories of alkali-metal ion in K_0.1_Na_0.3_Ni_0.2_Mn_0.8_O_2_ from the AIMD simulations. We find that sodium and potassium trajectories overlap, suggesting that potassium ions do not hinder sodium ions diffusion, since the latter can be where once occupied by the former. The radial distribution function (RDF) for the alkali ions are shown in Fig. 6d, and the RDF peaks are labeled corresponding to the labeled concentric circles in the lattice in Fig. 6e. The origin of the circles is chosen to be the center of an edge-sharing prism (marked with circled black star), as it is the lowest-energy alkali ion site in equilibrium. In Fig. 6d we see from K–K curve that K ions are more solid-like, with sharp RDF peaks, indicating they have much longer resident time on their respective sites than the time spent outside of site locations hopping. In Fig. 6e, sites forbidden to K ions around a central K ion are marked with black crosses. We see these are the nearest neighboring edge-sharing prism centers. This effect could be explained with the radius of potassium ions; large ions like potassium repel each other too strongly at this distance that such distribution is not possible. Sodium ions do not have as well defined peaks as the K-K case; they are more liquid-like. The time spent on residing on the sites is not significantly longer than the time hopping. This is another evidence showing Na ions are much more active than K ions. The full analysis of this AIMD data is presented in supplemental information. Here, we reach the conclusion that, when K and Na were both present, Na diffusion is not hindered by the presence of K ions, and Na ions diffuse much faster than K ions. We also verified it experimentally with galvanostatic intermittent titration technique (GITT) and found that with the presence of K ions, the Na ions have an average diffusion coefficient of 3 × 10^−10^ cm^2^ s^−1^, comparable to a few established results obtained without K ions (see Supplementary Fig. 12). The localization of the K^+^ ion resulted in a wide-spread distribution of “pillars”, found in the K-K RDF and in DFT structural optimization of K_0.1_Ni_0.2_Mn_0.8_O_2_, which created “homogenous” support for the transition metal layers as the sparse distribution of potassium ions made local depletion of these alkali-metal ions very unlikely (illustrated in Fig. 6f). This observation illustrates why 8% of the K^+^ ions were able to stabilize the layered structure even during the destructive P2-O2 phase transition. The detailed results of the density of states of the material, which verifies the charge transfer process, is put in the supplement information (as shown in Supplementary Fig. 13). It is worth noting that although we have suppressed the P2-O2 phase transition to achieve high capacity and long cycle life, the relatively low sodium content in the P2 cathode materials in general could still be a limiting factor for their commercial applications in sodium-ion batteries. Sodium supplementation through additives, pre-sodiated and metallic negative electrodes^50,51^ can be solutions to this issue.

To summarize, the pillar-beam structured O-P2-type K_0.4_[Ni_0.2_Mn_0.8_]O_2_ is successfully synthesized and used as a cathode material. It not only exhibits a high capacity of 194 mAh/g at 0.1 C, but also a high rate capacity of 84% at 1 C, and an 86% capacity retention after 500 cycles at 1 C within the voltage range from 1.5 to 4.2 V. Two essential characteristics of this material ensure its excellent electrochemical performance: (1) Only 8% of the pillar potassium ions are required to uphold the transition metal layers even with complete sodium extraction; (2) Only sodium ions participate in the intercalation reactions, which is realized by taking advantage of the differences in the thermodynamic and kinetic properties of K^+^ and Na^+^ ions. The findings obtained in this work inspire the search and design of improved positive electrode materials and the development of room temperature, long-lasting SIBs for use in large-scale energy storage systems.

## Methods

### Material synthesis

The series of materials of this work were prepared through a solid-state reaction method with the precursors K_2_CO_3_ (99%), Mn_2_O_3_, and NiO (99.5%). An excess of 5 mol% K_2_CO_3_ was used to compensate for the K loss during high-temperature synthesis. The starting materials were ground in an agate mortar and pressed into pellets under a pressure of 20 MPa. Then, the pellets were heated at 800 °C for 6 h, 900 °C for 6 h, 1000 °C for 10 h, and 1050 °C for 1 h in air. The heating rate was 5 °C/min. Afterwards, these pellets are cooled naturally. The color of the final products was black.

### X-ray diffraction

The XRD patterns of the pristine powders were measured on a Smartlab diffractometer (Rigaku) with Co radiation (Kα: 1.788920 Å), 40 kV, 40 mA, 0.02°/step, scan speed 4 s/step, and a range of 10.0–120.0°.

The in situ XRD patterns were collected from a modified 2032 coin-cell with a 15-μm thick Al foil as an X-ray window as well as a current collector for the cathode. The in situ X-ray measurement was performed on a Bruker D8 Advance diffractometer with 1 mm collimator and Cu-Kα radiation. The data were collected with scan-step size 0.025° and data collecting time of 3 s (2 h/scan). The corresponding electrochemical measurement was carried out using a Biologic SP300 potentiostat (BioLogic Science Instrument), controlled by EC-Lab (V10.19). The experimental coin cell was cycled between 1.5 and 4.2 V at a rate of C/15 for four cycles.

### X-ray absorption spectroscopy

The Mn and Ni K-edge XAFS spectra of the standards and electrode slides were collected at the beamline BL14W1 of the Shanghai Synchrotron Radiation Facility (SSRF) and beamline 17C1 and 44A1 of the National Synchrotron Radiation Research Center (NSRRC). The typical energies of the storage rings were 3.5 GeV and 3.0 GeV, and the electron currents were ~220 mA and ~500 mA in the top-up mode, respectively. The white light was monochromatized by a Si (111) double-crystal monochromator and calibrated with Mn foil (K-edge at 6539 eV) and Ni foil (K-edge at 8333 eV). Samples were positioned at 90° to the incident beam in the sample-holder and the XAFS spectra were recorded in transmission mode.

The XAFS data were analyzed using the Demeter software package. The spectra were normalized with Athena first, and then shell-by-shell fittings were performed with Artemis. The χ(k) function was Fourier transformed (FT) using k^3^ weighting, and all fittings were done in R-space. The amplitude reduction factor (S_0_^2^) was estimated to be 0.813 for Mn and 0.859 for Ni according to the fitting results of the manganese and nickel foil. The coordination parameters, including the coordination number, bond distance and Debye-Waller factor were allowed to vary and were obtained by fitting the experimental peaks with their theoretical amplitudes.

The oxygen K-edge near edge X-ray fine structure (NEXAFS) spectra of the electrode slides were collected at beamline BL10B in the National Synchrotron Radiation Laboratory (NSRL). The samples were kept in the total electron yield mode under an ultrahigh vacuum at 5 × 10^−5^ mbar during the experiment. The resolving power was E/ΔE = 1000 with the photon flux of 1 × 10^10^ photons s^−1^. Spectra were collected in 0.15 eV energy steps.

### Scanning transmission electron microscopy (STEM) imaging

A JEM-ARM200F STEM fitted with a double aberration-corrector for both probe-forming and imaging lenses was used to perform HAADF imaging, which was operated at 200 KV. The convergence angle was 25 mrad and the angular range of collected electrons for HAADF imaging was about 70–250 mrad. Atomic resolution STEM-EELS line scanning was performed vertically throughout the interfaces, providing an energy resolution of 0.4 eV.

### Differential electrochemical mass spectrometry (DEMS)

The DEMS experiments were performed with a commercial quadrupole mass spectrometer (Hiden Analytical) and a house-made Swagelok-type cell with K_0.4_Ni_0.2_Mn_0.8_O_2_ as cathode, sodium as anode and 1 M NaPF_6_ in ethylene carbonate (EC)/diethyl carbonate (DEC) (4:6 in volume) as electrolyte. The electrochemical test for DEMS is performed on a LAND battery testing system at room temperature. The carrier gas is high purity Ar-gas with a flow rate of 0.5 mL min^−1^.

### Electrochemistry

The working electrode was prepared by spreading a slurry of the active materials (75 wt.%), acetylene black (15 wt.%) and the polyvinylidene fluoride (10 wt.%) binder on Al foil. The working electrodes were dried at 120 °C in vacuum for 10 h. The electrolyte was 1 M NaPF_6_ in ethylene carbonate (EC)/diethyl carbonate (DEC) (4:6 in volume). The electrode mass loading is around ~5 mg/cm^2^. The coin-type (CR2032) cells were assembled with pure sodium foil as the counter electrode, and a glass fiber as the separator in an argon-filled glove box. The sodium storage performance in K_*x*_[Ni_*x*/2_Mn_1−*x*/2_]O_2_ | Na cells (*x* = 0.1, 0.2, 0.3, 0.4, 0.5, 0.6, 0.67) was tested, where sodium metal was used as the counter electrode and 1 M NaPF_6_ in 4:6 EC/DEC was used as the electrolyte. All assembled cells were left intact (OCV state) for 12 h before the first cycle in order to let the K^+^ and Na^+^ ion-exchange between electrolyte and K_*x*_[Ni_*x*/2_Mn_1−*x*/2_]O_2_ reach equilibrium. The charge and discharge measurements were carried out on a VMP-300 battery test system in the voltage range of 1.5–4.2 V at room temperature (1 C current rate corresponds to 120 mA/g).

### DFT calculations

First-principles calculations were performed using density functional theory (DFT) as implemented in the plane-wave-basis-set Vienna ab initio simulation package (VASP)^52^ with the projection augmented-wave scheme^53^. The Perdew–Burke–Ernzerhof (PBE) generalized gradient approximation (GGA) exchange-correlation functional with the rotationally invariant scheme of Hubbard-U correction^54^ was applied to calculate the total energies. The values of *U*_eff_ = 6.7 and 3.8 were employed for Ni and Mn, respectively, consistent with previous ab initio studies of their layered compounds^55–57^. The van der Waals interaction in structures at the charged state with a large amount of site vacancies was treated with nonlocal correlation functional vdW-DF-optB86^58^. Supercells containing 180–240 atoms were chosen depending on the composition of the alkali atoms. A cutoff energy of 520 eV was used for all calculations. The force exerted on each atom was ensured to be less than 0.01 eV/A for structural relaxation. The ionic diffusivity and conductivity were calculated using ab initio molecular dynamics (AIMD) as implemented in VASP. The simulations were performed on the canonical ensemble with a time step of 2 fs, and the temperature was initialized at 100 K and elevated to 400 K and 600 K with simulations over 40 ps for statistical analysis. The composition for AIMD is fixed to K_0.1_Na_0.3_Ni_0.2_Mn_0.8_O_2_. The radial distribution function analysis were processed with Python Materials Genomics package (pymatgen)^59^.

Structures with Ni/Mn and K/Na/vacancy orderings are pre-screened using Ewald electrostatic energy criterion in pymatgen^59^ followed by DFT geometry optimizations. Ni/Mn ordering are taken from the calculation of as-synthesized composition of K_0.4_Ni_0.2_Mn_0.8_O_2_ and kept fixed in the other calculations, assuming Ni/Mn cannot diffuse in the transition-metal layer when the cathode is charged or discharged. For K_*x*_Ni_0.2_Mn_0.8_O_2_ (*x* = 0.1, 0.2, and 0.4), 40 lowest-energy structures are chosen for DFT optimization for each composition. For Na_*x*_K_0.1_Ni_0.2_Mn_0.8_O_2_ (*x* = 0.1, 0.2, 0.3, and 0.5) and Na_0.2_K_0.2_Ni_0.2_Mn_0.8_O_2_, 20 structures in each composition are chosen for DFT optimization.

## Supplementary information

Supplementary Information

## Data Availability

The data that support the findings of this study are available from the corresponding author upon reasonable request.
