# Supplementary material for: Pillar-beam structures prevent layered cathode materials from destructive phase transitions
Source: Nat Commun. 2021 Jan 4;12:13. doi: 10.1038/s41467-020-20169-1 (PMC7782780; doi:10.1038/s41467-020-20169-1)
Supplement: Supplementary file 1 — Supplementary Information [file 41467_2020_20169_MOESM1_ESM.pdf]

Pillar-beam structures prevent layered cathode materials from destructive phase transitions

Yuesheng Wang[1] *†, Zimin Feng[1] †, Peixin Cui[2] †, Wen Zhu[1†], Yue Gong[3], Marc-André Girard[1], Gilles Lajoie[1], Julie Trottier[1], Qinghua Zhang[3], Lin Gu[3] *, Yan Wang[4*], Wenhua Zuo[5], Yong Yang[5], John B. Goodenough[6], Karim Zaghib[1] *

1. Center of Excellence in Transportation Electrification and Energy Storage Hydro Québec 1800 Boulevard Lionel-Boulet, Varennes, Québec, J3X 1S1 Canada

2. Key Laboratory of Soil Environment and Pollution Remediation, Institute of Soil Science, Chinese Academy of Sciences, Nanjing, Jiangsu, 210008, China

3. Laboratory of Advanced Materials and Electron Microscopy, Beijing National Laboratory for Condensed Matter Physics, Institute of Physics, Chinese Academy of Sciences, Beijing, 100190, China

4. Advanced Materials Lab, Samsung Research America, Cambridge, MA, 02183, USA

5. State Key Laboratory for Physical Chemistry of Solid Surfaces, and Department of Chemistry, College of Chemistry and Chemical Engineering, Xiamen University, Xiamen 361005, People's Republic of China

6. The University of Texas at Austin, Austin, Texas, 78712, United States

† These authors contributed equally to this work.

Correspondence to:

Yuesheng Wang (Email:Wang.Yuesheng@ireq.ca)

Lin Gu (Email: l.gu@iphy.ac.cn);

Yan Wang (Email: eric.wangyan@samsung.com);

Karim Zaghib (Email: Zaghib.Karim@ireq.ca)

Supplementary Notes

Analysis of the AIMD data:

Ab-initio molecular dynamics (AIMD) simulation was performed to investigate the diffusion of alkali metal ions in the P2 phase. Fig. 6c shows trajectories of alkali metal ion from the AIMD simulations. We see sodium and potassium trajectories do overlap, suggesting that potassium ions do not hinder sodium ions diffusion, since the latter can be where once occupied by the former. The radial distribution function (RDF) for the alkali ions are shown in Fig. 6d, and the RDF peaks are labeled corresponding to the labeled circles in the lattice in Fig. 6e, where the origin of the circles is chosen to be a center of edge-sharing prism (marked with circled black star), as it is the lowest-energy alkali ion site in equilibrium.

There are some interesting features revealed by the AIMD simulations. Firstly, the K-K RDF has well-defined peaks, while Na-Na RDF is much smoother. This means potassium ions are more solid-like, staying at the same site for longer time, hop between sites quickly and then stay at new sites for another while. In Fig. 6e the RDF peak locations and possible sites for potassium ions match well, as the site centers locates right on the circles, except for minor discrepancies that are likely due to lattice distortion by the movements and distributions of alkali metal ions. We note, however, that the nearest neighbor edge-sharing prism center is forbidden (marked with black crosses; 3.1 Å from origin), and the nearest possible occupation is a surface-sharing prism center, whose equilibrium occupation is smaller. We ascribe this phenomenon to the large radius of potassium ions, that potassium ions at neighboring edge-sharing prism centers repel each other so strongly that they cannot show up together at normal conditions.

Sodium ions, on the other hand, does not have such restrictions, and the first RDF peak appear at 2 Å. With smaller radius, a sodium ion located at origin could well be approached by another sodium ion locating at the nearest neighbor site in spite of the fact that this neighboring site is a surface-sharing prism center with less occupation at equilibrium. This peak 1 has a relatively lower amplitude; this can be explained by its peak location (2 Å). In fact, the distance between the origin and the nearest neighbor site is 1.75 Å, slightly less than 2 Å (Na RDF peak 1). This indicates that the sodium ions still have enough radius to repel each other if they get as close as the neighboring sites. This leads to an instability, since sodium ions not able to locate at the centers of the prism cannot be as stable as those do locate at the centers. And this instability in turn leads to shorter resident time and

hence lower amplitude of peak 1. Except for this peak 1, no other peaks are as well-defined. This liquid-like feature indicates that sodium ions are very active in diffusing. When they don't stay at specific sites long enough, they cannot show sharp peaks in RDF. The sites at neighboring edge-sharing prism centers (black crosses in Fig. 6e) don't seem to be forbidden to them sodium ions, but with a following peak from the same sites as peak 2 of K-K curve in addition to their high activity, the occupation at these sites are not shown as a peak, but just a rising slope at 3.1 Å.

The RDF of sodium ions around a central potassium ion shows still other features. We see at 3.1 Å there is also a rising slope, meaning the neighboring edge-sharing prism centers are not strictly forbidden. But apparently there is greater repel from the central potassium ion that this probability is smaller than that of Na-Na case. Besides the common peak at 3.5 Å marked as peak 2, it has one more peak at 4.5 Å, marked as peak 3 in Fig. 6d and whose possible contributing sites are marked with blue stars in Fig. 6e. This is in sharp contrast to K-K case, where all sites at 4.5 Å are rarely occupied. Again, this could be explained with the fact that sodium ions have smaller radius. The distance between closest peak 2 sites and peak 3 sites is 3.1 Å, the same as the distance between the origin and the K-K forbidden sites, so when peak 2 sites are occupied, peak 3 sites are much less likely to be occupied by K ions, while sodium ions, void of such strong K-K repel, can safely locate there. Given such low probability of peak 3 sites occupations by K ions in the K-K case, we don't see a good reason why they must be forbidden. With longer simulation time or larger supercell, we expect to see larger value of K-K RDF at 4.5 Å.

The largest peak in RDF is at 8.5 Å for the K-K case. But the peak location is already more than half of the size of the supercell of choice that we believe this effect to be artificial.

Analysis of the Jahn-Teller distortion:

In the region of Mn K-edge XANES spectra, the two tiny pre-edge peaks at 6541.5 and 6543.2 eV arise from the 1s to 3d-hybridized-4p transition. According to the ligand field theory, the peak above 6544 eV is attributed to the 1s to t_{1u^*} transition, and the two tiny pre-edge peaks at ~ 6541 and 6543 eV arise from the 1s to e_{g^*} and t_{2g}, associated to a quadrupolar transition and a dipolar transition, respectively [Phys. Rev. B. 2004, 70, 033104]. The dipolar transition is forbidden in the normal octahedra but allowed due to the structure disorder. Jahn-Teller distortion occurs in Mn^{3+} ([Ar]$3d^4$), but not in Mn^{4+}([Ar]$3d^3$), and decreases the symmetry of the MnO_6 octahedra, which increases the intensity of the

pre-edge peaks [Adv. Funct. Mater. 2014, 24, 5112-5118]. Meanwhile, the white-line peak is split into two peaks due to the different bond lengths of Mn-O resulted from Jahn-Teller distortion. According to the Natoli's rule and the bond lengths of Mn-O in table S2, the shoulder peak should appear ~ 8.0 eV ahead the white-line peak, which could be observed in the spectra. Overall, the Jahn-Teller distortion enhances the pre-edge peaks and splits the white-line peak. These spectral features show up at lower voltages during the charge-discharge cycles, where the portion of Mn^{3+} is increased. Thus, the appearance of the spectral features, an indicator of Jahn-Teller distortions, matches well with the charge/discharge state. Conversely, there is no pre-edge in the Ni K-edge XANES region, as Ni^{2+}([Ar]$3d^8$) does not cause Jahn-Teller effect and the Ni-O bonds in NiO_6 octahedral share the same bond length.

Supplementary Tables

Supplementary Table 1a Lattice parameters determined from the Rietveld refinement for the $K_{0.4}Ni_{0.2}Mn_{0.8}O_2$ and $K_{0.3}N_{i0.15}Mn_{0.85}O_2$.

Items	Parameters	$K_{0.4}Ni_{0.2}Mn_{0.8}O_2$	$K_{0.3}N_{i0.15}Mn_{0.85}O_2$	
Space group		CMCM	C/2M	
Crystal system		Orthogonal	Monoclinic	
Cell parameter	a (Å)	2.8865	5.1187	
	b (Å)	5.0017	2.8603	
	c (Å)	12.7908	7.1810	
	α (º)	90	90	
	β (º)	90	102.8	
	γ (º)	90	90	
	V (Å^3)	190.007	105.012	
R_{wp}		0.88%	1.42%	
R_p		0.68%	0.93%	
Chi^2		0.8219	2.1521	
SOF	4c	0.0451	4i	0.315
	4c	0.3875		

Supplementary Table 1b Atomic positions and occupancies determined from the Rietveld refinement for the $K_{0.4}Ni_{0.2}Mn_{0.8}O_2$ and $K_{0.3}N_{i0.15}Mn_{0.85}O_2$.

$K_{0.4}Ni_{0.2}Mn_{0.8}O_2$ (O-P2 phase)						
**Site**	**wyckoff**	**x**	**y**	**z**	**occ.**	**temperture factor**
Ni	4a	0	0	0	0.2105	0.0026
Mn	4a	0	0	0	0.78095	0.0014
K(1)	4c	0	0. 0000	0.75	0.0451	0.0167
K(2)	4c	0	0.333 (0)	0.75	0.3875	3.018
O	8f	0	0.309(3)	0.0696	1	0.7(2)
**$K_{0.3}Ni_{0.15}Mn_{0.85}O_2$ (P3 phase)**						
**Site**	**wyckoff**	**x**	**y**	**z**	**Occ.**	**temperture factor**
K	4i	-0.1026	0.5	0.3318	0.305	0.2
Mn1	4i	-0.18561	0.5	0.6662	0.8	2.811
Ni1	4i	-0.18561	0.5	0.6662	0.2	3.158
Mn2	2a	0	0	0	0.8	0.813
Ni2	2a	0	0	0	0.2	1.283
O1	4i	-0.4294	0.5	0.3856	1	3.2
O2	4i	0.2285	0	0.0018	1	4.2
O3	4i	-0.0024	0.5	0.1346	1	0.116

In this study, our focus is the O-P2 phase with composition of $K_{0.4}Ni_{0.2}Mn_{0.8}O_2$, therefore, the temperatures used were optimized for obtaining the O-P2 phase with minimum impurity, not optimised for P3 phase formation (compositions K<0.3). Using the same temperature schedule to heat the samples with different compositions only serves the purpose of direct comparison of the final powders.

Supplementary Table 2. The Valence of Mn at the different statement of Charge-Discharge

Sample	Absorption edge (eV)	Valence		Sample	Absorption edge (eV)	Valence
2.35V 1st disch.	6554.8	3.29		4.2V 1st ch.	6558.0	3.95
1.5V 1st disch.	6553.9	3.10		3.4V 2nd disch.	6557.1	3.76
2.2V 1st ch.	6554.4	3.21		2.35V 2nd disch.	6556.0	3.54
2.7V 1st ch.	6554.9	3.31		1.5V 2nd disch.	6554.0	3.12
3.3V 1st ch.	6556.8	3.70		OCV	6556.5	3.64
				pristine	6556.6	3.66

Note: ch.=charge; disch.=discharge

Supplementary Table 3a. EXAFS fitting parameters at the Mn K-edge for various samples

Sample	Shell	CN^a	R(Å)^b	σ^2(Å^2)^c	ΔE_0(eV)^d	R factor
**Mn K edge**						
Mn_1st-disch. 2.35 V	Mn-O	3.6	1.86	0.0056	-9.2	0.0002
	Mn-O	2.2	1.99	0.0056		
	Mn-Mn/Ni	7.7	2.89	0.0100		
Mn-1st-disch. 1.5 V	Mn-O	3.5	1.86	0.0060	-7.5	0.0012
	Mn-O	2.4	1.99	0.0060		
	Mn-Mn/Ni	7.5	2.88	0.0088		
Mn_1st-ch. 2.2 V	Mn-O	3.5	1.86	0.0058	-9.1	0.0005
	Mn-O	2.4	1.99	0.0058		
	Mn-Mn/Ni	8.1	2.89	0.0088		
Mn_1st-ch. 2.7 V	Mn-O	3.6	1.86	0.0063	-9.6	0.0003
	Mn-O	2.3	1.99	0.0063		
	Mn-Mn/Ni	7.9	2.88	0.0085		
Mn_1st-ch. 3.26 V	Mn-O	3.3	1.86	0.0057	-8.0	0.0004
	Mn-O	2.5	1.99	0.0057		
	Mn-Mn/Ni	7.6	2.88	0.0073		
Mn_1st-ch. 4.2 V	Mn-O	2.7	1.83	0.0059	-9.4	0.0016
	Mn-O	3.2	1.95	0.0059		
	Mn-Mn/Ni	7.1	2.85	0.0064		
Mn_2nd-disch. 3.4 V	Mn-O	3.4	1.86	0.0049	-8.0	0.0004
	Mn-O	2.3	1.99	0.0049		
	Mn-Mn/Ni	8.0	2.88	0.0072		
Mn_2nd-disch. 2.3 V	Mn-O	3.6	1.86	0.0039	-9.9	0.0006
	Mn-O	2.2	1.99	0.0039		
	Mn-Mn/Ni	7.7	2.89	0.0086		
Mn_2nd-disch. 1.5 V	Mn-O	3.5	1.86	0.0070	-9.2	0.0004
	Mn-O	2.3	1.99	0.0070		
	Mn-Mn/Ni	7.3	2.89	0.0099		
Mn-OCV	Mn-O	3.2	1.86	0.0047	-8.4	0.0010
	Mn-O	2.5	1.98	0.0047		
	Mn-Mn/Ni	7.6	2.88	0.0068		
Mn-Pristine	Mn-O	3.4	1.86	0.0062	-4.6	0.0003
	Mn-O	2.6	1.99	0.0062		
	Mn-Mn/Ni	8.1	2.88	0.0072		

Supplementary Table 3b. EXAFS fitting parameters at the Ni K-edge for various samples

Sample	Shell	*CN*[a]	*R*(Å)[b]	σ^2(Å^2)[c]	ΔE_0(eV)[d]	*R* factor
**Ni K-edge**						
Ni_1st-disch. 2.35 V	Ni-O	5.8	2.06	0.0055	-1.5	0.00003
	Ni-Mn/Ni	9.0	2.95	0.0072		
Ni-1st-disch. 1.5 V	Ni-O	6.0	2.06	0.0058	-2.1	0.00001
	Ni-Mn/Ni	8.9	2.95	0.0070		
Ni_1st-ch. 2.2 V	Ni-O	5.9	2.06	0.0055	-2.0	0.00002
	Ni-Mn/Ni	9.2	2.95	0.0069		
Ni_1st-ch. 2.7 V	Ni-O	5.8	2.06	0.0059	-1.8	0.00003
	Ni-Mn/Ni	8.8	2.95	0.0068		
Ni_1st-ch. 3.26 V	Ni-O	6.1	2.06	0.0056	-2.5	0.00001
	Ni-Mn/Ni	9.3	2.95	0.0065		
Ni_1st-ch. 4.2 V	Ni-O	5.7	2.06	0.0053	-1.8	0.00003
	Ni-Mn/Ni	9.2	2.96	0.0077		
Ni_2nd-disch. 3.4 V	Ni-O	5.8	2.06	0.0054	-2.1	0.00001
	Ni-Mn/Ni	9.1	2.95	0.0069		
Ni_2nd-disch. 2.3 V	Ni-O	5.9	2.07	0.0054	-1.7	0.00001
	Ni-Mn/Ni	9.2	2.96	0.0066		
Ni_2nd-disch. 1.5 V	Ni-O	5.6	2.07	0.0056	-1.6	0.00001
	Ni-Mn/Ni	8.6	2.96	0.0068		
Ni_OCV	Ni-O	5.9	2.06	0.0056	-2.3	0.00001
	Ni-Mn/Ni	8.9	2.95	0.0068		
Ni_Pristine	Ni-O	6.0	2.06	0.0056	-2.4	0.00001
	Ni-Mn/Ni	9.1	2.95	0.0068		

[aC]*N*: coordination numbers; [b]*R*: bond distance; [c]σ^2: Debye-Waller factors; [d] ΔE_0: the inner potential correction. *R* factor: goodness of fit. S_0^2 was set to 0.813 for Mn and 0.859 for Ni, according to the experimental EXAFS fit of Mn and Ni foil references by fixing CN as the known crystallographic value.

Supplementary Table 4. The lattice parameters "c/2" of $K_{0.4}Ni_{0.2}Mn_{0.8}O_2$ electrode at the different stages of Charge-Discharge

c/2 (Å) by	XRD	DFT	STEM
$K_{0.4}Ni_{0.2}Mn_{0.8}O_2$	6.397	6.412(P2)	6.430
$K_{0.2}Na_{0.2}Ni_{0.2}Mn_{0.8}O_2$	5.709	6.101(P2)<br>6.197(O2)	5.606
$K_{0.1}Na_{0.7}Ni_{0.2}Mn_{0.8}O_2$	5.352	5.428(P2)	5.321
$K_{0.08}Ni_{0.2}Mn_{0.8}O_2$	4.761	5.664(P2)<br>6.033(O2)	4.859

Our DFT optimized lattice parameters agree with experimental results very well in the cases of $K_{0.4}Ni_{0.2}Mn_{0.8}O_2$ and $K_{0.1}Na_{0.7}Ni_{0.2}Mn_{0.8}O_2$. There are noticeable mismatches in compositions with small amount of Na/K especially in $K_{0.2}$ and $K_{0.08}$ cases. This is due to the following reasons. 1) mismatch in the K composition. The corresponding calculated composition is actually $K_{0.1}Ni_{0.2}Mn_{0.8}O_2$ due to the limited size of simulated supercell. An actual smaller K concentration would cause more significant layer collapsing therefore a smaller c-axis in experiments. 2) DFT error in the case of significant vacant alkaline metal layer. It is well known that DFT with GGA has difficulty handle weak correlated layered structures with dispersion (van der Waals) forces; therefore the calculated lattice parameter is larger in this case with only a small amount of K/Na in the alkaline layer. We applied vdW corrections in the DFT calculations by using the nonlocal correlation functional vdW-DF-optB86 [Ref : J. Klimeš, D. R. Bowler, and A. Michaelides, J. Phys.: Cond. Matt. 22, 022201 (2010).] but apparently this correction is still not perfect. Further studies maybe needed to optimize the use of different vdW-DF corrections in order to get the perfect match.

It is worth noting that our DFT calculations predict a significant smaller lattice parameter for the low-energy O2 phase (5.67, closer to the experiments) than the high-energy P2 (6.03) in $K_{0.1}Ni_{0.2}Mn_{0.8}O_2$. Indeed, we observed a phase change from P2 to O2 at the small K concentration in experiments. In $K_{0.2}Ni_{0.2}Mn_{0.8}O_2$ the low-energy P2 phase has a smaller lattice parameter (6.10, closer to experiment) than the high-energy O2 phase (6.20). Despite the calculated values do not match perfectly with experiments in these two cases, the discovered trend in the change of the lattice parameters with phase transitions agrees with experimental observations very well.

Supplementary Figures

K distribution

Firstly, we examined batteries with sodium metal as the counter electrode. We performed the EDS on the washed separator and sodium. On the sodium surface, we could not detect any potassium.

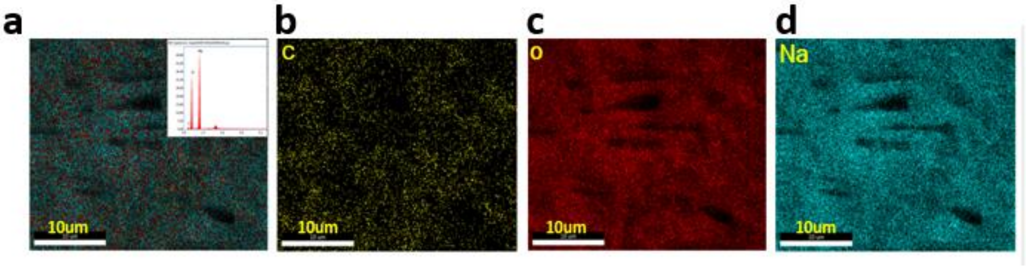

Supplementary Figure 1. Chemical mapping of the sodium surface in batteries with sodium metal as counter electrode. a EDX spectrum image **b** C **c** O **d** Na distribution.

On the separator, we could detect potassium indicating that potassium is located in the electrolyte.

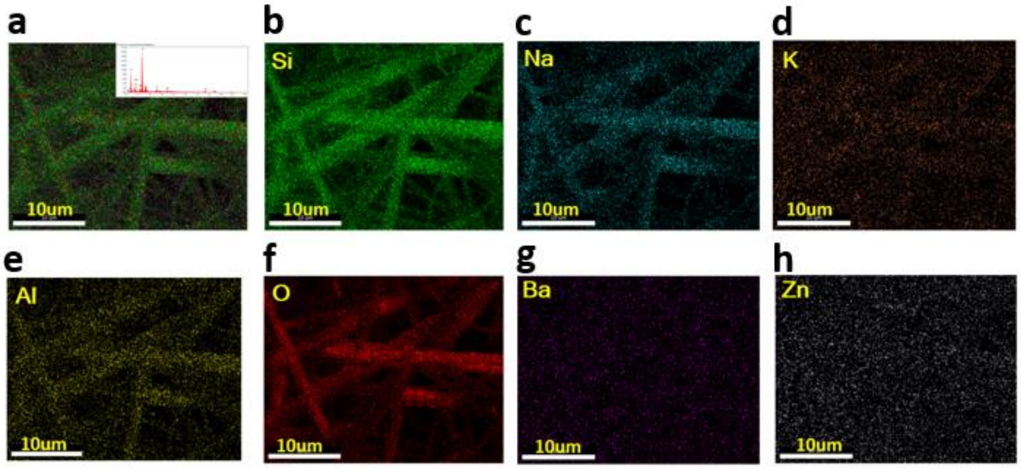

Supplementary Figure 2. Chemical mapping of the separator in batteries with sodium metal as counter electrode. a EDX spectrum image **b** Si **c** Na **d** K **e** Al **f** O **g** Ba **h** Zn

Secondly, we have analysed the batteries with hard carbon as the counter electrode. Our EDS results are as follows,

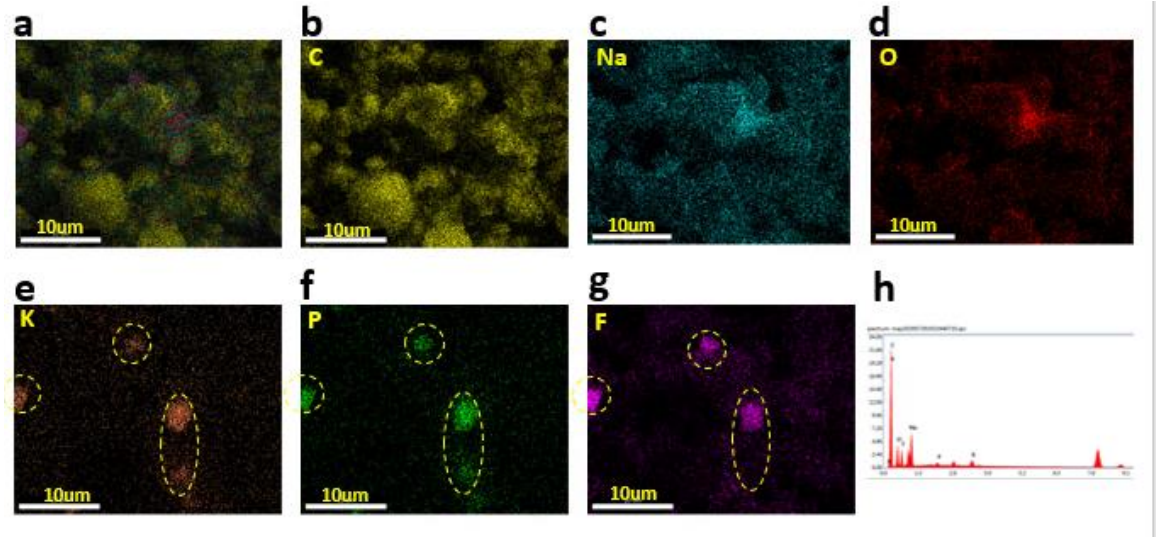

Supplementary Figure 3. Chemical mapping of the hard carbon in batteries with hard carbon as counter electrode. a EDX spectrum Image **b** C **c** Na **d** O **e** K **f** P **g** F **h** EDX spectra

From above images, we can see sodium distributed roughly uniformly on the hard Carbon. Particles containing K, P and F are also observed, suggesting potassium existed in the stable form of KPF_6.

The separator from the cell with hard carbon anode was also examined and the results are as follow:

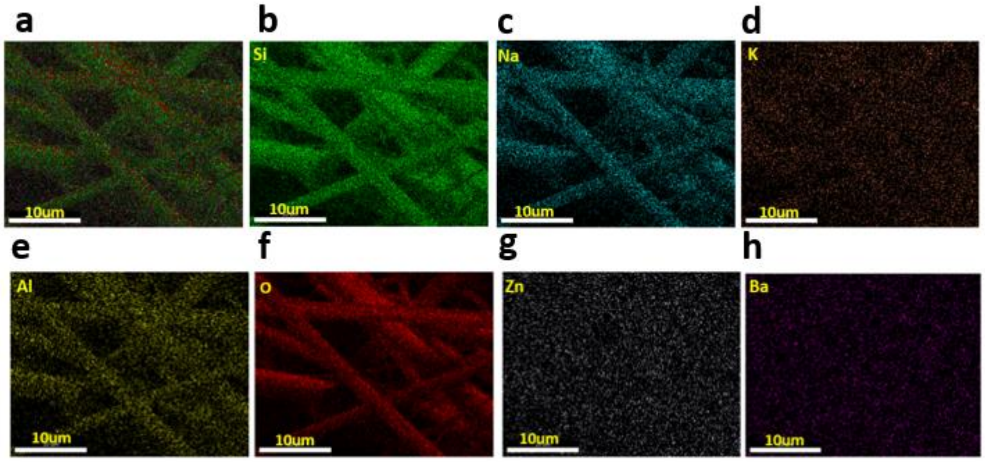

Supplementary Figure 4. Chemical mapping of the separator in batteries with hard carbon as counter electrode. a EDX spectrum image **b** Si **c** Na **d** K **e** Al **f** O **g** Zn **h** Ba

From the above image, we can see that potassium ions are uniformly distributed in the separator, which again suggests the existence of K in the electrolyte as in the case of cell with sodium metal anode.

At the same time, we did the ICP for Sodium metal, separator and hard carbon. The results are as follows:

ICP results of Hard carbon, Hard carbon separator, sodium metal and sodium-metal-separator

Name	K (mg/L)	Na (mg/L)
Hard Carbon	0.014	0.052
Hard Carbon-separator	13.6	/
Sodium Metal	0.008	/
Sodium-Metal-separator	7.92	/

From the above table, we can see most of the potassium stay in the electrolyte. The ratio of (potassium in sodium/hard carbon) / (potassium in separator) is around 1/900. These results are consistent with EDS results.

All above evidences suggests that there is ion exchange between potassium and sodium. Ion exchanged potassium became KPF_6 in the electrolyte.

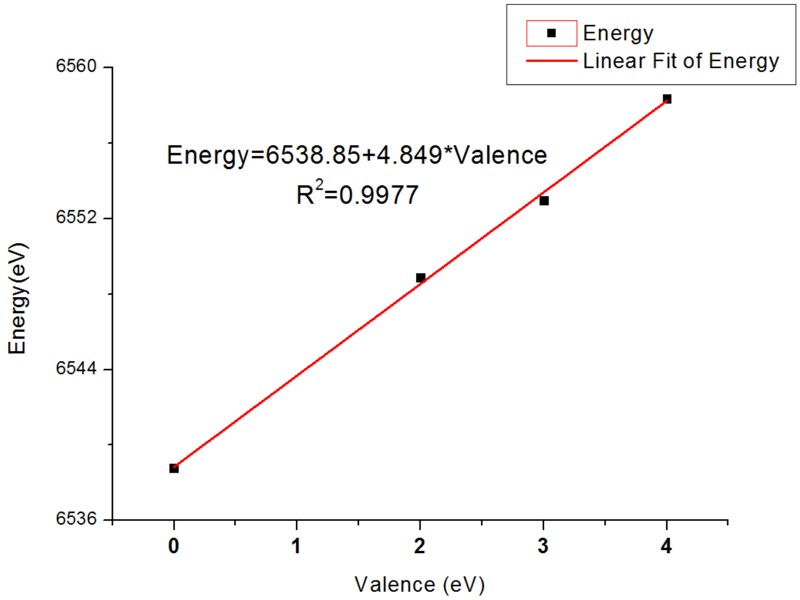

Supplementary Figure 5. The image of linear fit of energy and valence for Mn standards.

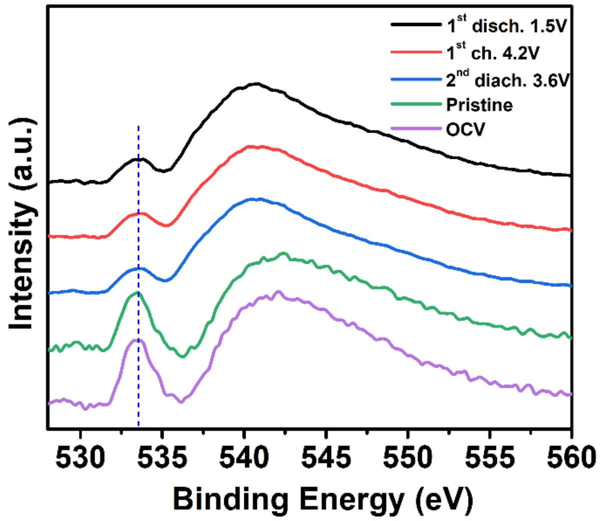

Supplementary Figure 6. O K-edge NEXAFS of different States.

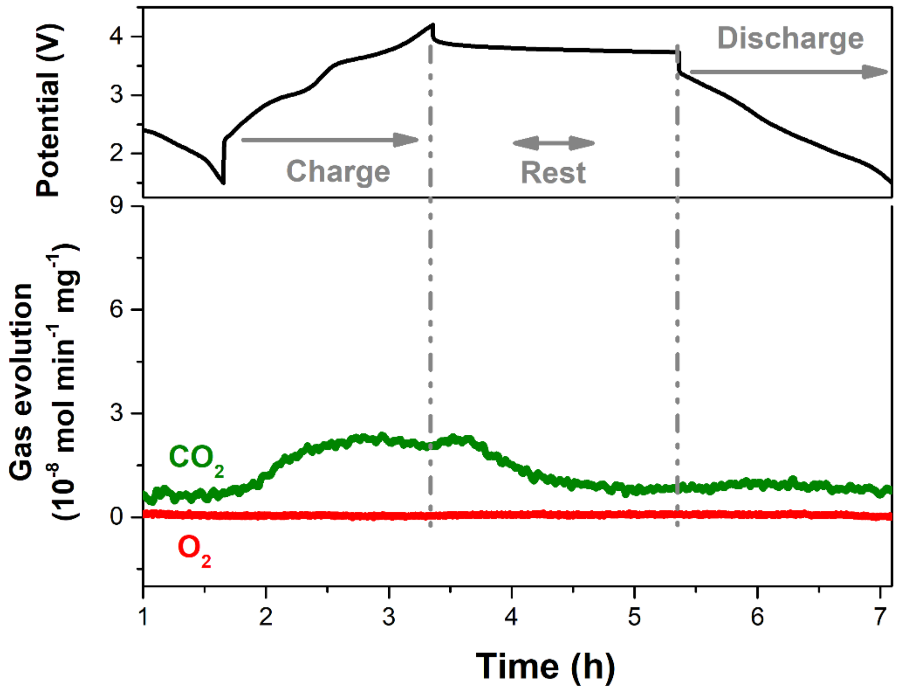

Supplementary Figure 7. The discharge–charge voltage profile of the sodium ion battery with $K_{0.4}Ni_{0.2}Mn_{0.8}O_2$/$NaPF_6$ EC: DMC/ Na at the current rate of 0.5C and the corresponding differential electrochemical mass spectrometry (DEMS) result

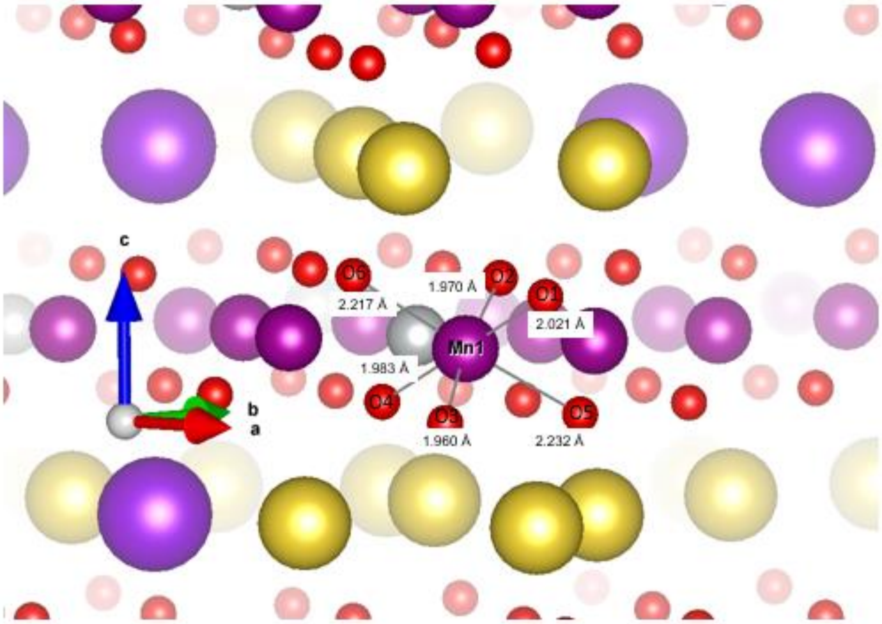

Supplementary Figure 8. Jahn−Teller active Mn^{3+} cations in the $K_{0.1}Na_{0.5}Ni_{0.2}Mn_{0.8}O_2$ (marked as Mn1).

We have confirmed the Mn^{3+} J-T distortion in the DFT calculated structure of $K_{0.1}Na_{0.5}Ni_{0.2}Mn_{0.8}O_2$ (Fig S4), which shows the MnO_6 octahedron with Mn^{3+} is dramatically distorted as compared to Mn^{4+}. The calculated Mn-O bonds are listed here (2.22 A, 1.98 A, 1.97 A, 1.96A, 2.02 A and 2.23 A) to confirm it is J-T distorted.

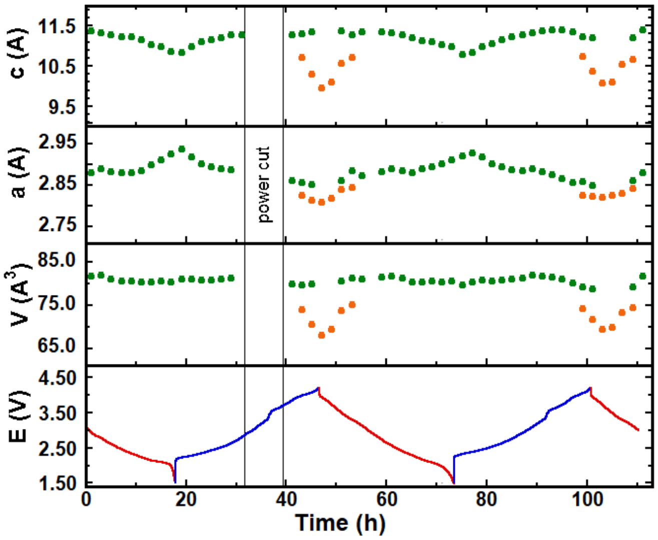

Supplementary Figure 9. Lattice parameter variations during first two cycles. Red: discharge; blue: charge; green: H-P2 phase; orange: O2 phase. The missing data is due to power cut.

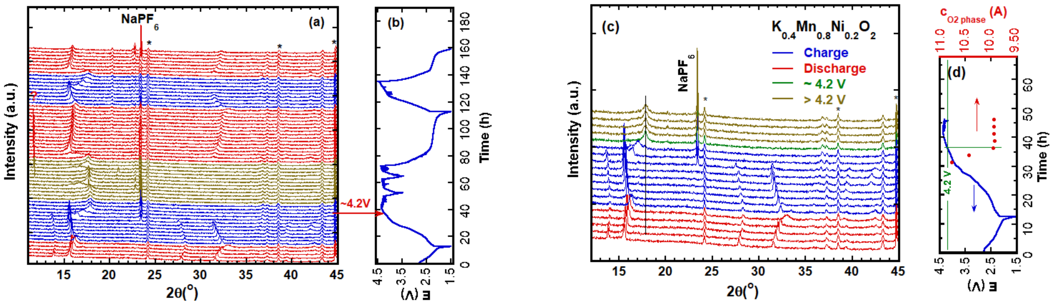

Supplementary Figure 10. In-situ XRD patterns of $K_{0.4}Ni_{0.2}Mn_{0.8}O_2$ electrode during electrochemical operation. a Evolution of XRD patterns of the first two cycles at C/15 between 1.5 and ~ 4.5 V; **b** Voltage-time curve corresponds to **a; c** XRD zoomed on the first cycle; **d** Voltage-time curve corresponds to **c** and lattice parameter c of the O2 phase.

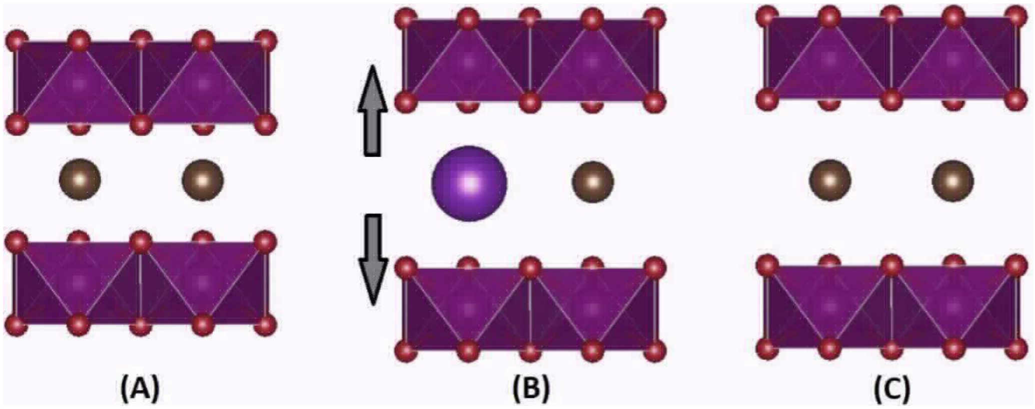

Supplementary Figure 11. Cathode structures with only Na ions **(A),** expanded cathode structure with K ions **(B)** and expanded cathode structure with K ions replaced by Na ions **(C).**

Effect of K ions in reducing the Na intercalation potential.

High enough voltages is a necessary condition for OERs to occur, thus reducing the voltage will effectively help preventing OERs to happen. The K ions, with a radius larger than that of Na ions, repel the transition metal layers, making it impossible for the latter to provide optimum inter-layer distance for Na, so that when Na ions intercalates, the voltage it results is lower than the case with smaller (optimum) inter-layer distance between transition metal layers. DFT calculations are performed to estimate the amount of this voltage change with and without K ions, considering the three scenarios:

A. Na ions intercalating into $Na_zK_{0.08}Ni_{0.2}Mn_{0.8}O_2$. The Na intercalation potential is calculated by the difference in total energies of all involved compounds, V_A=-[E($Na_{z+y}K_{0.08}Ni_{0.2}Mn_{0.8}O_2$)-E($Na_zK_{0.08}Ni_{0.2}Mn_{0.8}O_2$)-E($Na_y$)]/(y*e). From our calculation (see below) we have V_A=[5.439-E(Na)]/e where E(Na) is the normalized Na metal energy.

B. Without K ions, Na intercalating into $Na_{z+0.08}Ni_{0.2}Mn_{0.8}O_2$. This scenario could give the Na intercalation voltage without the effect of K. V_B=-[E($Na_{z+y+0.08}Ni_{0.2}Mn_{0.8}O_2$)-E($Na_{z+0.08}Ni_{0.2}Mn_{0.8}O_2$)-E($Na_y$)]/(y*e). In this case the calculated V_B=[5.602-E(Na)]/e which is 0.16 V higher than V_A.

C. To understand why $V_A<V_B$, we ran another calculation without Na but manually fixed the inter-layer distance of the transition metal layers to that of K-containing $Na_{z(+y)}K_{0.08}Ni_{0.2}Mn_{0.8}O_2$ (keeping the expanded cathode lattice from A but replacing all K with Na). This time we calculate V_C=-[E($Na_{z+y+0.08}Ni_{0.2}Mn_{0.8}O_2$)$_{expanded}$-E($Na_{z+0.08}Ni_{0.2}Mn_{0.8}O_2$)$_{expanded}$-E($Na_y$)]/(y*e) and we get V_C=[5.381-E(Na)]/e.

The results indicate that V_A is very close to V_C (only differ by 0.058 V), but both of them are lower than V_B by about 0.2 V. The lowered voltage in the case of A (with K) and C (without K but with expanded lattice) as compared to B (without K) is mainly due to the stretching of the transition metal layers by K ions. These calculations demonstrate that the doped K ions significantly reduced the Na intercalation voltage, therefore suppressing the high voltage OER effect.

In the above calculations, structures are modeled with supercells containing 60 formula-units, and 6 K and 6 Na for case A, 12 Na for case B and C, respectively. Before Na intercalation, the structures contain 6 K and 5 Na for case A, and 11 Na for case B and C, respectively. Thus, we effectively took y+z=0.1 and y=0.0166. And the K0.08 composition is approximated with K0.1 due to the limited supercell size in DFT calculations. O2 phase is assumed, because this is the high voltage phase where OER usually happens.

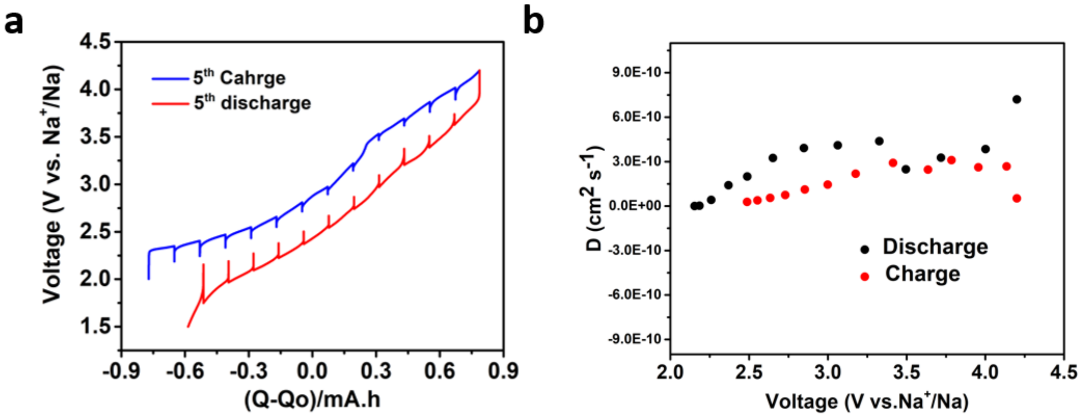

Supplementary Figure 12. a GITT curves of the $K_{0.2}Mn_{0.8}Ni_{0.2}O_2$ electrode; **b** the sodium-ion chemical diffusion coefficients calculated from GITT at 0.1C for 2 h working time, followed by 3 h relaxation of $K_{0.4}Ni_{0.2}Mn_{0.8}O_2$ and the measured sodium ion diffusion coefficients as a function of working voltage during 1.5- 4.2V.

Analysis of the density of states of Mn and Ni

It is interesting to look at the changes in the density of states (DOS) of Mn and Ni during the charge cycles. Three configurations are chosen for the calculation of DOS, namely, $K_{0.1}Na_{0.5}Mn_{0.8}Ni_{0.2}O_2$, $K_{0.1}Na_{0.3}Mn_{0.8}Ni_{0.2}O_2$ and $K_{0.1}Mn_{0.8}Ni_{0.2}O_2$. They correspond to the charge state voltage from low to high. The DOS plots for these configurations are shown in Fig. S13a-c. Solid purple and cyan peaks represent Mn and Ni *d*-orbitals, respectively.

Comparing Fig.S13a ($K_{0.1}Na_{0.5}Mn_{0.8}Ni_{0.2}O_2$) and Fig.S13b ($K_{0.1}Na_{0.3}Mn_{0.8}Ni_{0.2}O_2$), we see that one of the *d*-orbitals of Mn originally occupied in $K_{0.1}Na_{0.5}Mn_{0.8}Ni_{0.2}O_2$ (in the spin-up top panel, the cyan peak right below the Fermi level) becomes empty in $K_{0.1}Na_{0.3}Mn_{0.8}Ni_{0.2}O_2$. This indicates that, during cell charge, Mn changes valence before Ni. And from Fig.S13b ($K_{0.1}Na_{0.3}Mn_{0.8}Ni_{0.2}O_2$) to Fig.S13c ($K_{0.1}Mn_{0.8}Ni_{0.2}O_2$), the *d*-orbital that was occupied in Fig.S13b (in the spin-up top panel, the purple peak below the Fermi level) became empty in Fig.S13c too, indicating that Ni changed valence at higher voltage (when Na are fully extracted).

These results are well consistent with our XANES results, that at lower voltages, Ni stays inactive, while at higher voltages Ni involves in charge transfer processes too.

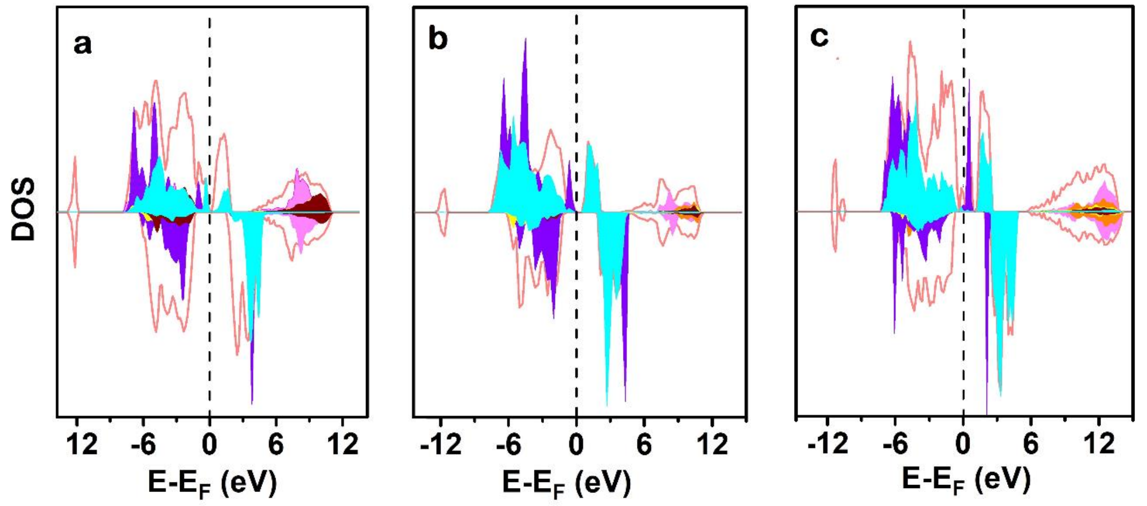

Supplementary Figure 13. Density of states (DOS) of cathode material for **a** $K_{0.1}Na_{0.5}Mn_{0.8}Ni_{0.2}O_2$, **b** $K_{0.1}Na_{0.3}Mn_{0.8}Ni_{0.2}O_2$ and **c** $K_{0.1}Mn_{0.8}Ni_{0.2}O_2$.

E_F represent the Fermi level. The red curves are for the total DOS. The solid pink, orange and purple peaks are for *s*-, *p*- and *d*-orbitals of Ni; the solid yellow, brown and cyan peaks are for *s*-, *p*- and *d*-orbitals of Mn. In particular, the relative positions of the *d*-orbital peaks of Mn (cyan) and Ni (purple) versus the Fermi level illustrate the charge transfer processes during the cell operation.
